# Nano-Brushes of Alcohols Grafted onto Cellulose Nanocrystals for Reinforcing Poly(Butylene Succinate): Impact of Alcohol Chain Length on Interfacial Adhesion

**DOI:** 10.3390/polym12010095

**Published:** 2020-01-04

**Authors:** Hatem Abushammala

**Affiliations:** Fraunhofer Institute for Wood Research (WKI), Bienroder Weg 54E, 38108 Braunschweig, Germany; hatem.abushammala@wki.fraunhofer.de

**Keywords:** cellulose, nanocrystals, modification, poly(butylene succinate), interfacial adhesion

## Abstract

Despite the many interesting properties of cellulose nanocrystals (CNCs), their hydrophilicity is one of the main challenges for their processing with hydrophobic polymers and matrices. To overcome this challenge, this paper describes the preparation of brush-like CNCs with tailored surface properties by grafting alcohols of different chain lengths onto their surfaces. Ethanol, 1-butanol, 1-hexanol, and 1-octanol were grafted on the CNC surface using 2,4-toluene diisocyanate (TDI) as a linker. The CNCs were characterized for their structural, morphological, surface, and thermal properties. Because of the grafting, the water contact angle of the CNCs significantly increased from 32° to up to 120°, which was dependent on the chain length of the grafted alcohol. The thermal stability of the CNCs was also improved, mainly as a result of the reaction of TDI with the CNC hydroxyl groups. Later, the CNCs were used to reinforce films of poly(butylene succinate) (PBS), which were then characterized using dynamic mechanical analysis (DMA) and thermogravimetric analysis (TGA). An increase of up to two-fold in the storage modulus was observed using DMA, which was dependent on the chain length of the grafted alcohol. However, no change in the glass transition temperature or degradation temperature of PBS was detected. This approach is proved efficient for tailoring the surface properties of CNCs towards excellent interfacial adhesion in their composites.

## 1. Introduction

Cellulose nanocrystals (CNCs), a form of nanocellulose, are rod-like nanoparticles with a thickness of 3–10 nm and a length of a few hundred nanometers [1]. They can be extracted from cellulose and wood using a variety of chemicals and techniques [2,3,4]. CNCs have inherited many of the properties of cellulose such as mechanical strength, biocompatibility, and hydrophilicity [5]. Due to their nanosize and shape, CNCs have a high surface area of up to 500 m^2^/g and can form liquid crystalline structures [6,7]. For many years, CNCs were mainly used to mechanically reinforce thermoplastic and thermoset matrices such as polyethylene, poly(lactic acid), polyurethanes, and others [8,9]. A major challenge there is overcoming the hydrophilicity of CNCs as they tend in most cases to agglomerate instead of dispersing within the hydrophobic matrix [10]. Other than the use of compatibilizers to improve CNC/matrix interfacial adhesion [11], a wide range of chemical modifications have been explored to reduce the hydrophilicity of CNCs. These chemical modifications included relatively simple procedures such as acetylation, silylation, and carbanilation, or grafting bulky hydrophobic moieties onto the CNC surface [12,13,14,15].

Grafting hydrophobic groups on the CNC surface may require the use of a linker. Among many, 2,4-toluene diisocyanate (TDI) is one of the most commonly used due to the significant difference in the reactivity of its isocyanates [16]. The ortho-isocyanate groups are 5–10 times less reactive than the para ones due to the steric hindrance from the neighboring methyl group [17,18]. The difference in reactivity is also dependent on the reaction conditions. High reaction temperature, for instance, leads to the reaction of both isocyanates with CNC hydroxyls or to self-polymerization [19]. The reaction of TDI with CNCs was first reported by Habibi and Dufrense in 2008 after being performed for decades on cellulose [16,20]. Since then, the reaction has more often been used to graft functional molecules and polymers onto nanocellulose surfaces [21]. The reaction was only recently optimized to react all the CNC surface hydroxyls with TDI molecules, most of which have their ortho-isocyanates available for a further reaction with certain functional molecules [22,23].

**Figure 1:**
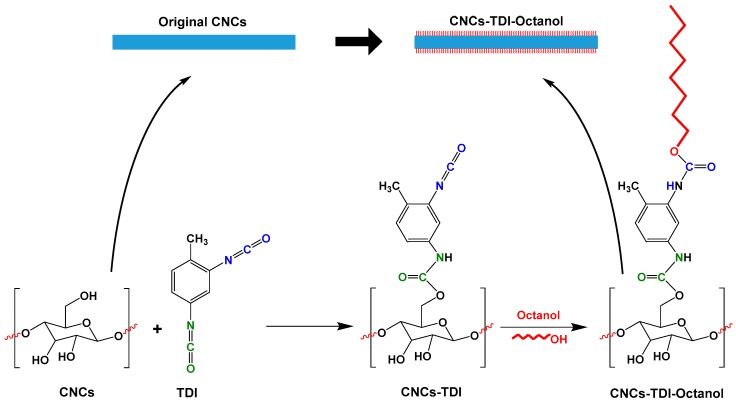
The proposed approach to tailor the hydrophilicity of cellulose nanocrystals (CNCs) by grafting alcohols of different chain lengths on the CNC surface using 2,4-toluene diisocyanate (TDI) as a linker.

With the help of TDI, this work explores the possibility of tailoring the hydrophilicity of CNCs by grafting alcohol groups of different chain lengths onto the CNC surface (Figure 1). Following this approach, the more-reactive para-isocyanate groups of TDI will selectively react with the surface hydroxyl groups of CNCs. The ortho-isocyanate groups will then react with the hydroxyl groups of the different alcohols (ethanol, 1-butanol, 1-hexanol, and 1-octanol), which will ultimately lead to the formation of brush-like CNCs. As a result, this approach could allow the preparation of CNCs with a wide range of surface properties and consequently excellent miscibility with a variety of polymers and matrices including poly(butylene succinate) (PBS).

PBS is a biodegradable aliphatic polyester, which is produced by the polycondensation of 1,4-butanediol and succinic acid [24]. It was invented in the early 1990s with similar properties to poly(ethylene) and poly(propylene) [25], and has shown great potential in many applications including automotive and packaging industries and tissue engineering [26,27,28]. PBS has been processed with CNCs to improve its mechanical, thermal, and barrier properties, its biodegradability, and to control its morphology and crystallization [29,30,31,32]. Different approaches have been used to improve the interfacial adhesion between CNCs and PBS. Fortunati et al. used a surfactant while Luduena et al. used poly(ethylene glycol) as a compatibilizer to achieve that goal [33,34]. Phthalic anhydride was also used as a compatibilizer [35,36]. Interfacial adhesion has been also improved by chemical modification of CNCs (acetylation for instance) [37] and by PBS polymerization is situ [38,39]. This paper proposes that processing PBS with CNCs of tailored surface properties would expand the possibilities to fine-tune its properties and could ultimately foster the use of PBS in many applications.

## 2. Materials and Methods

### 2.1. Materials

The CNC suspension (solid content of 10.4% *w*/*w*) was purchased from the University of Maine, which was prepared using sulfuric acid. Acetone, toluene, triethylamine (TEA), ethanol, 1-butanol, 1-hexanol, 1-octanol, and chloroform were purchased from VWR (Darmstadt, Germany) and stored under an A3 or A4 molecular sieve. The molecular sieves were purchased from Carl Roth (Karlsruhe, Germany) and regenerated before use. TCI Chemicals (Eschborn, Germany) provided 2,4-toluene diisocyanate. Bio-based poly(butylene succinate) was purchased from PTTMCC (Bangkok, Thailand) under the commercial name BioPBS ^TM^.

### 2.2. Grafting of Alcohols onto the Surface of the CNCs

The CNCs were reacted with TDI following the method of Habibi and Dufresne after minor modifications [20] and using the optimum conditions recommended by Abushammala [23]. Of the 10.4% CNC suspension (equivalent to 1.0 g of dried CNCs (6.2 mmol)), 9.6 g was solvent-exchanged from water to anhydrous acetone using a washing/precipitation procedure (three times), then to anhydrous toluene using the same procedure (twice). The precipitation was performed using a Sigma 3–16P centrifuge (g-force of 4472, 5000 rpm for 30 min) (Sigma Laborzentrifugen, Osterode am Harz, Germany). After the final washing with toluene, the precipitated CNCs were transferred to a 100 mL round-bottom flask using 46.3 mL of anhydrous toluene. 3.3 g of 2,4-TDI (equivalent to 18.6 mmol) and 3.0 mL of triethylamine (as a catalyst) were then added to the reaction flask. The reaction proceeded at 35 °C for 24 h in a moisture-free environment (under nitrogen). The reaction mixture was then centrifuged to collect the TDI-carbamated CNCs (CNCs-TDI) from the unreacted TDI and triethylamine. The CNCs-TDI were then washed three times with anhydrous toluene before being transferred to 50 mL of anhydrous ethanol and stirred for 24 h at room temperature to allow complete grafting of the alcohol on the CNC surface. The CNCs were then collected by centrifugation and transferred using acetone to a pre-weighed aluminum dish. The CNCs were dried at 60 °C under vacuum to a constant mass. The mass of the dried CNCs was then determined to estimate the mass yield of the reaction. To assure reproducibility, the reaction was performed in duplicate. The reaction was repeated using 1-butanol, 1-hexanol, and 1-octanol instead of ethanol. The produced CNCs are referred to in the paper as CNCs-TDI-Eth, CNCs-TDI-But, CNCs-TDI-Hex, and CNCs-TDI-Oct, respectively. A sample of the CNCs after TDI-carbamation and before alcohol grafting was prepared and referred to as CNCs-TDI.

### 2.3. Processing of the Alcohol-Grafted CNCs with PBS

Of the alcohol-grafted CNCs, 0.25 g was dispersed in 20 mL of chloroform using the ultrasonicator UW2200 (10% of 2200 W, 20 s) (Bandelin Electronic, Berlin, Germany). To that, 4.75 g of PBS was added and mixed until complete dissolution was achieved. The homogeneous mixture was transferred to an aluminum dish and heated gradually to 135 °C to remove the chloroform and to melt the PBS. The samples were kept at that temperature for 24 h then cooled down to room temperature. Following this procedure, films with a thickness of ca. 1 mm containing 5% (*w*/*w*) of CNCs were produced and are referred to in the manuscript as PBS + CNCs-TDI, PBS + CNCs-TDI-Eth, PBS + CNCs-TDI-But, PBS + CNCs-TDI-Hex, and PBS + CNCs-TDI-Oct. It was not possible to prepare films using the original CNCs because of their hydrophilicity.

### 2.4. Characterization of the Alcohol-Grafted CNCs

#### 2.4.1. Elemental Analysis Using SEM-EDX

The change in the elemental composition of the CNCs after TDI-carbamation and the following surface grafting of the alcohols was determined using a ZEISS GeminiSEM Crossbeam 340 scanning electron microscope (ZEISS, Oberkochen, Germany), which was equipped with an X-MaxN EDX detector (Oxford Instruments, Abingdon, UK). The samples in the form of powder were pressed in a mold to obtain discs (diameter: 1 cm) of smooth surfaces. The measurements were performed using a voltage of 10 kV.

#### 2.4.2. Structural Characterization Using Fourier Transform Infrared (FT-IR)

Samples of the CNCs before and after modification were characterized using a Tensor 27 FT-IR spectrometer (Bruker, Billerica, MA, USA) in the 750–4000 cm^−1^ range (resolution of 2 cm^−1^) using the ATR transmission mode. The spectra were obtained as the average of 64 scans and analyzed using OPUS 6.5 software.

#### 2.4.3. Morphological Characterization Using Atomic Force Microscopy (AFM)

A drop of a diluted suspension (10^−4^%) of the original and modified CNCs was deposited on a fresh mica surface then kept to air-dry overnight. The original CNC suspension was in water while the modified CNCs were suspended in chloroform. The surface was imaged in the tapping mode using the atomic force microscope Agilent 5500 (Keysight Technologies, Santa Rosa, CA, USA). The silicon tips PPP-NCH (Wetzler, Nanoandmore, Germany) were used which had a resonance frequency of ca. 350 kHz and a spring constant of ca. 50 N.m^−1^. The thickness of the CNCs was determined based on a sample size of 100 particles using the Gwyddion software (version 2.26).

#### 2.4.4. Water Contact Angle Measurements

Powder samples of the CNCs before and after modification were pressed in a mold to obtain discs (diameter: 1 cm) of smooth surfaces. The water contact angle was determined by placing a water droplet of a volume of 15 µL on each surface using OCA20 equipment (DataPhysics Instruments GmbH, Filderstadt, Germany). The standard tangent procedure was followed to determine the contact angle. The measurements were done in triplicate to assure reproducibility.

#### 2.4.5. Thermal Stability Using Thermogravimetric Analysis (TGA)

The thermal stability of the CNCs before and after surface modification was investigated using the thermogravimetric analyzer TGA/DSC-1-STARe (Mettler Toledo, Giessen, Germany). Around 10 mg of each sample was heated from 25 to 1000 °C in a nitrogen environment using a heating rate of 10 °C/min. The measurements were performed in duplicate. The degradation temperature (T_d_) was determined as the temperature at the maximum first derivative while the onset temperature (T_o_) was determined using the tangent method.

### 2.5. Impact of the Alcohol-Grafted CNCs on the Morphological and Thermomechanical Properties of PBS

#### 2.5.1. Dispersibility of CNCs within PBS

To investigate the dispersibility of CNCs within the PBS matrix, the surface of the PBS samples with and without the alcohol-grafted CNCs was coated using amorphous carbon and imaged using a ZEISS GeminiSEM Crossbeam 340 scanning electron microscope (ZEISS, Oberkochen, Germany). The images were collected using an electron energy of 2 kV, working distance of 5 mm, and magnification of 10,000 times.

#### 2.5.2. Thermomechanical Properties using a Dynamic Mechanical Analyzer (DMA)

PBS samples with and without the alcohol-grafted CNCs were cut to pieces with the dimensions 30 mm × 8 mm × 1 mm and tested using Tritec 2000 DMA (Triton technology Ltd., Nottinghamshire, UK). Dual cantilever bending mode was applied using a displacement of 0.05 mm, which is within the linear viscoelastic range, and a frequency of 1 Hz. The samples were cooled down to −60 °C using liquid nitrogen before heated up to 100 °C at a rate of 4 °C/min.

#### 2.5.3. Thermal Stability Using Thermogravimetric Analysis (TGA)

To study the impact of the CNC addition on the thermal stability of PBS, around 10 mg samples were analyzed using the thermogravimetric analyzer TGA/DSC-1-STARe (Mettler Toledo, Giessen, Germany) following the procedure in 2.4.5.

## 3. Results and Discussion

The surface properties of CNCs have a crucial role in controlling their other properties such as mechanical reinforcement capabilities, biocompatibility, biodegradability, dispersibility, and thermal stability [40]. As a result, they directly impact their potential in many applications such as water purification and tissue engineering [41,42]. The surface energy of CNCs is dependent on the carbon backbone of the cellulose chains but more importantly on the surface hydroxyl groups, which are more often subjected to chemical and physical treatments [10,43]. Small degrees of chemical modifications have been shown to have a major impact on the surface properties of cellulose [44].

Earlier, it has been shown by the author that the CNCs used in this paper have ca. 7.5% of their total hydroxyl groups available on the surface for a possible chemical modification [22]. The author has also optimized the reaction of these CNCs with TDI towards reacting all surface hydroxyl groups selectively with the para isocyanate of TDI [23]. It was evident that the reaction temperature had a negative impact on the para/ortho selectivity of the isocyanate groups while the molar ratio of TDI/CNCs improved it. Overall, a maximum para/ortho selectivity was obtained using 35 °C and a molar ratio of 3. Under these reaction conditions (which are used in this paper), all of the surface hydroxyls of the CNCs are reacted with TDI and 93% of the ortho-isocyanates are available for the following reaction such as the grafting of alcohols in this paper [23]. It is important to mention that the reaction of TDI with CNCs was also optimized to make sure that the reaction does not take place inside the CNCs. Otherwise, the hydrogen bonding network in there would be disrupted and their reinforcing capabilities would be undermined [45].

The reaction of CNCs with TDI and the following grafting of the alcohols can be monitored by the change in the elemental composition of the CNCs (Table 1). Upon TDI-carbamation, the carbon and nitrogen content of the CNCs increased because of the attachment of the TDI molecules to the CNC surface through their para isocyanates. For the same reason, a 25% increase in the CNC mass was determined. The following reaction of the alcohols with the ortho-isocyanates of TDI led to a further increase in the carbon content, which was dependent on the chain length of the alcohol. A smaller increase in the mass yield was measured as the alcohols used in this study have a smaller molar mass (a maximum of 130.2 g/mol for 1-octanol) compared to TDI (174.2 g/mol). The increase in the mass yield was clearly dependent on the chain length of the alcohol. To confirm the efficacy of TDI-carbamation and the following alcohol grafting, the theoretical mass yield of both reactions was calculated assuming that all CNC surface hydroxyls reacted with TDI and that all the free ortho-isocyanate groups of TDI reacted then with the alcohol. The results showed that the experimental mass yield is in a close agreement to the theoretical yield indicating the efficiency of both TDI-carbamation and alcohol grafting on the CNC surface.

**Figure 2:**
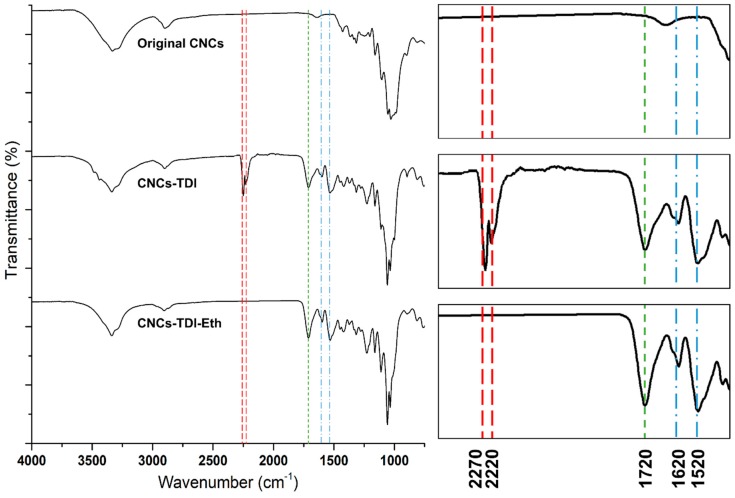
FT-IR spectra monitoring the structural changes in the CNCs upon TDI-carbamation and the following grafting of alcohols. The spectra on the right side are zoomed in a range of 1400–2400 cm^−1^.

The TDI-carbamation and alcohol-grafting reactions were also monitored using FT-IR (Figure 2). The original CNCs showed the typical vibration bands of cellulose at 1110 cm^−1^ for glycosidic bond vibration, at 1430 cm^−1^ for C-H deformation vibration, at 1640 cm^−1^ for adsorbed water vibration, at 2890 cm^−1^ for C-H stretching vibration, and at 3300 cm^−1^ for O-H stretching vibration [46]. TDI-carbamation of the CNCs was evident by the appearance of the bands of the aromatic ring vibration at 1520 and 1620 cm^−1^, the stretching vibration of the carbonyl group of the urethane bond at 1720 cm^−1^, and the asymmetric isocyanate vibration at 2220 and 2270 cm^−1^ as TDI reacted with the surface of the CNCs forming a urethane bond. Upon grafting the alcohols on the CNC surface through the free isocyanates, the isocyanate vibration bands disappeared confirming their reaction with the alcohols. The intensity of the carbonyl stretching vibration band at 1720 cm^−1^ increased as grafting led to a further formation of urethane bonds.

**Figure 3:**
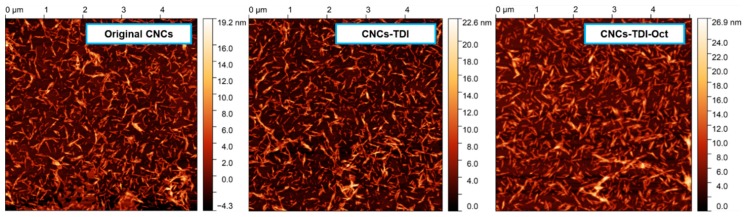
AFM height images of the CNCs before and after surface modification indicating no significant change in the morphology.

Any possible change in the morphology of the CNCs upon carbamation and grafting was investigated using AFM (Figure 3). The height images showed no significant change in the shape of the particles in general. No significant agglomeration, crosslinking, or even thickening was also observed. The thickness of CNCs-TDI and CNCs-TDI-Oct was respectively 8.0 ± 1.0 nm and 8.7 ± 1.3, which are slightly higher than that for the original CNCs (thickness of 7.0 ± 1.6 nm). This means that the thickness of the graft is not more than 1–2 nm.

**Figure 4:**
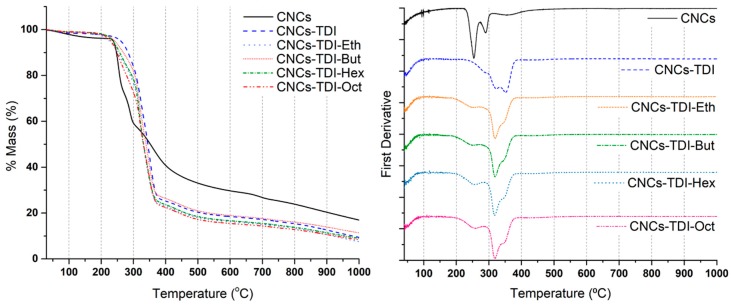
Thermogravimetric analysis (TGA) and DTG results of the CNCs before and after surface modification showing the significant change in thermal stability.

The impact of TDI-carbamation and alcohol-grafting on the thermal stability of the CNCs were investigated using TGA (Figure 4 and Table 2). A two-stage degradation was observed for the original CNCs, which is in line with the typical thermal behavior of the CNCs prepared using sulfuric acid. The degradation temperature was around 250 °C, which is lower than the typical degradation temperature of wood cellulose (350–400 °C) [47]. That is a result of the increase in the free end chains due to cellulose depolymerization during the production of the CNCs [48]. A relatively high char fraction of around 18% is a result of the sulfate groups acting as flame retardants [49]. These values are all in agreement with the reported values in the literature for CNCs [50]. Upon TDI-carbamation, the thermal stability of the CNCs changed significantly in the form of an increase in the degradation temperature to 351 °C and a decrease in the char fraction to 9.3%. The increase in the degradation temperature and onset is a result of derivatizing the less stable CNC hydroxyl groups by the TDI molecules [51] and the complete degradation of the surface TDI molecules could be the reason for the lower char fraction [52]. It is important to clarify that the two-stage degradation of the carbamated CNCs does not mean that the degradation mechanisms of the CNCs and carbamated CNCs are similar. Alcohol grafting made the degradation more heterogeneous in the form of a three-stage degradation. The second and third degradation stages for all alcohol-grafted CNCs took place at the same temperature as the first and second degradation stages of the carbamated CNCs (320 and 351 °C, respectively). This may imply that the first stage in the degradation of the alcohol-grafted CNCs is a result of the degradation of the grafted alcohol. Overall, the degradation temperature of the CNCs upon alcohol grafting decreased from 351 to around 320 °C while the char fraction did not change significantly.

**Figure 5:**
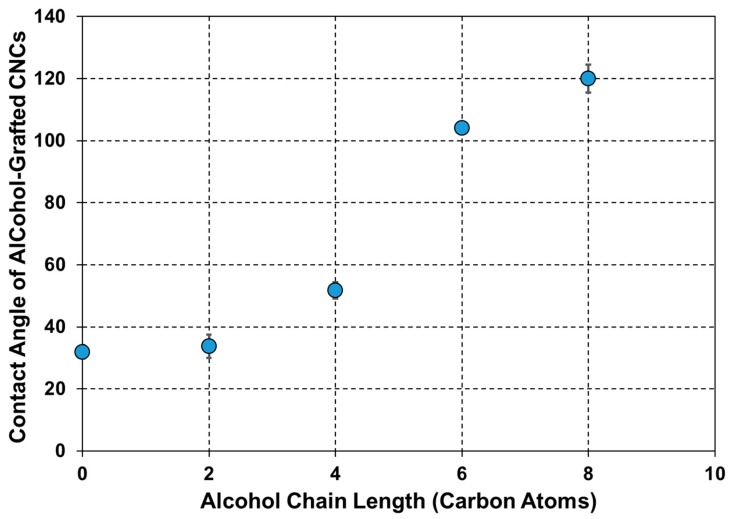
The dependence of the contact angle of the modified CNCs on the chain length of the alcohols grafted onto their surfaces.

The previous characterization techniques confirmed the successful grafting of the four alcohols on the CNC surface using TDI as a linker and assessed its impact on the structural, morphological, and thermal properties of the CNCs. Since the main goal of this grafting is to tailor the hydrophilicity of CNCs towards improving their interfacial adhesion with hydrophobic matrices, the water contact angle of the alcohol-grafted CNCs was determined (Figure 5). The contact angle of the original CNCs was 32° ± 2, which is in agreement with the values reported in the literature [3,53]. Upon carbamation, it was not possible to measure the water contact angle of CNCs-TDI as the water droplet reacted immediately with the free isocyanates. Upon alcohol grafting, the contact angle increased to a value of up to 120° ± 5, which was dependent on the chain length of the grafted alcohol. This value is higher than the water contact angle of PBS itself (77° ± 3). In more detail, CNCs-TDI-Eth did not show a significant increase in the contact angle as the hydrophobicity introduced by the ethyl groups was balanced by the hydrophilicity of the newly formed urethane bonds as they introduced highly polar C=O, C-O, C-N, and N-H bonds [54,55]. Increasing the chain length caused the hydrophobicity of the alkyl groups to become prevailing. A maximum change in the contact angle from 52° ± 3 to 104° ± 1 when the grafted alcohol was 1-hexanol instead of 1-butanol implies the evolution of an alkyl-based hydrophobic shell around the CNCs. This shell was more perfected with increasing the chain length from six to eight but with a less significant increase in contact angle from 104° ± 1 to 120° ± 5. A plateau could be expected at a maximum contact angle of 140° if the curve is extrapolated.

**Figure 6:**
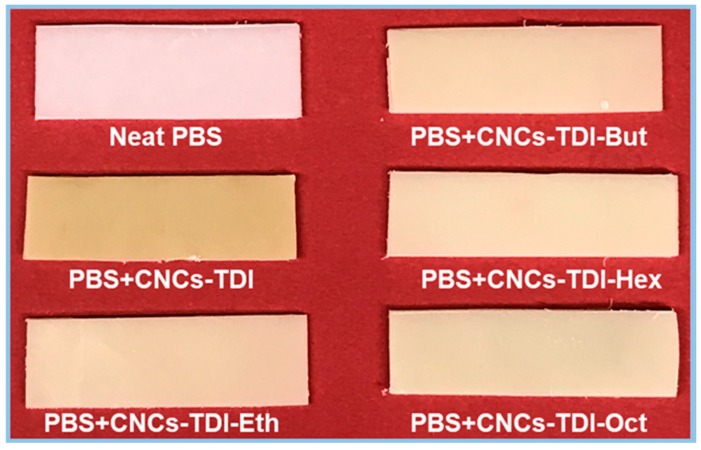
The change in the color of poly(butylene succinate) (PBS) upon processing with the modified CNCs.

**Figure 7:**
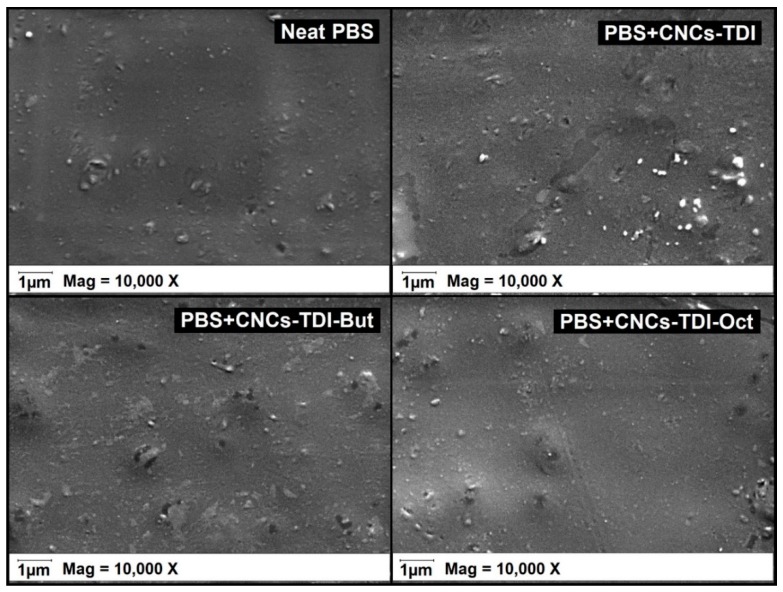
SEM images of PBS upon the addition of the modified CNCs indicating no CNC agglomeration. The small spheres were observed in all samples even the neat PBS. They are possibly a result of sample cutting.

It is clear now that it is possible to tailor the surface properties of CNCs by grafting alcohols of different chain lengths onto their surfaces. The impact of this procedure on the interfacial adhesion between CNCs and PBS was investigated for films of 5% (*w*/*w*) CNCs in PBS (Figure 6 and Figure 7). No agglomeration of the CNCs can be observed visually or using SEM, indicating a good dispersibility of the CNCs within the PBS matrix, which was not possible to achieve using the original CNCs. However, a clear change in the color of PBS from white to off-white was observed, which was more significant for the PBS reinforced with CNCs-TDI. This change in color was not a result of any thermal degradation of the CNCs or PBS as the TGA results (Figure 4 and Table 3) showed that both materials (CNCs and PBS, separately) started to degrade at temperatures far higher than the temperature used for making the films (135 °C). No chemical reactions can be foreseen as a reason for this color change as the CNCs and PBS are expected to be chemically inert to each other. The only possible interaction between them is expected to be physical, in the form of adhesion. It is possible, therefore, that the crystallization of PBS in the presence of CNCs may have resulted in the formation of different spherulites and consequently a change in optical properties [56]. The impact of the alcohol-grafted CNCs on the crystallization kinetics of PBS will be thoroughly discussed in a follow-up paper.

**Figure 8:**
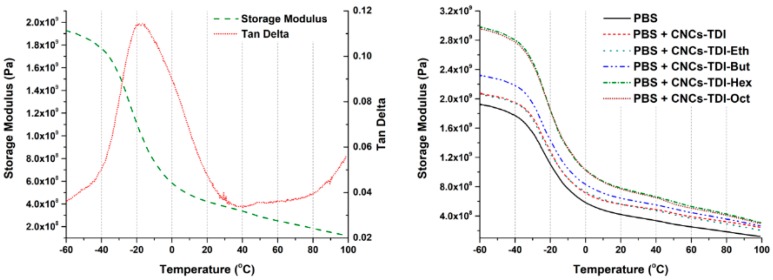
The dynamic mechanical analysis (DMA) results of the neat and reinforced PBS samples showing the glass transition temperature of PBS at around −17 °C (**Left**) and the effect of the surface modification of CNCs on their reinforcement capabilities (**Right**).

**Figure 9:**
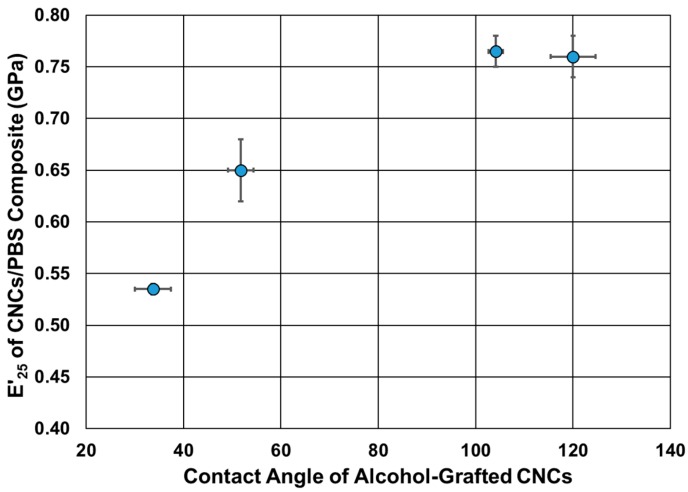
The relationship between the contact angle of the alcohol-grafted CNCs and their capabilities to reinforce PBS.

The impact of the alcohol-grafted CNCs on the thermomechanical behavior of PBS was studied using DMA (Figure 8). The storage modulus and glass transition temperature values of neat PBS are in agreement with the values in the literature [57] (Table 3). Upon the addition of the alcohol-grafted CNCs, an increase of up to two-fold in the storage modulus was observed, which was dependent on the chain length of the grafted alcohol. The storage modulus at 25 °C increased from 0.37 GPa to 0.52 GPa upon the addition of CNCs-TDI. Almost the same increase was observed upon the addition of CNCs-TDI-Eth. This indicates that the grafting of ethanol did not significantly change the surface properties of the CNCs as shown earlier in Figure 5. The modulus increased to 0.65 GPa using CNCs-TDI-But and reached a plateau at 0.77 GPa using either CNCs-TDI-Hex or CNCs-TDI-Oct. These results could be explained considering the contact angle of the alcohol-grafted CNCs (Figure 9). CNCs-TDI-Eth and CNCs-TDI-But were not hydrophobic enough to obtain an optimum interfacial adhesion with PBS. However, the surface properties of PBS seem to match those for CNCs-TDI-Hex and CNCs-TDI-Oct. Therefore, an alky chain length of six to eight seems to be needed for the formation of a hydrophobic shell around the CNCs and consequently improving the interfacial adhesion between PBS and the CNCs. In contrary to the storage modulus, the glass transition temperature (determined using DMA) and degradation temperature (determined using TGA) of PBS were not affected by the addition of any of these CNCs (Table 3).

In summary, grafting alcohols of different chain lengths on the surface of CNCs with the help of TDI is an efficient approach to tailor the surface properties of CNCs and to allow their processing with hydrophobic matrices such as PBS. The grafting does not only influence the properties of the CNCs but also those of their composites. To confirm the versatility of this approach, the interfacial adhesion of these CNCs with other hydrophobic matrices will be covered in a future publication.

## 4. Conclusions

Nano-brushes of different alcohols grafted on the surface of CNCs were investigated as an approach to control the hydrophilicity of CNCs. They were prepared by grafting ethanol, 1-butanol, 1-hexanol, and 1-octanol on the CNC surface using TDI as a linker. The reaction was confirmed gravimetrically and using elemental analysis and FT-IR. AFM showed no significant change in the shape and size of the CNCs upon grafting. The water contact angle of the CNCs increased significantly from 32° to up to 120° and was directly dependent on the chain length of the grafted alcohol. The increase in the contact angle implied the evolution of a hydrophobic alkyl-based shell around the CNCs. The produced CNCs were then used to reinforce the hydrophobic polymer PBS. Using DMA, an increase of up to two-fold in the storage modulus was measured, which was dependent on the chain length of the grafted alcohol and the corresponding CNC contact angle. No significant change in the glass transition temperature or degradation temperature of PBS was observed. Overall, grafting alcohols of different chain lengths onto the CNC surface was efficient for tailoring the surface properties of the CNCs and improving their miscibility with PBS.

## Figures and Tables

**Table 1:** Monitoring the change in the elemental composition of CNCs before and after surface modification and the resulting increase in mass yield.

Sample	C (%)	O (%)	N (%)	S (%)	Na (%)	Mass Yield	Theoritical Yield
CNCs	43.7	55.3	0.0	0.5	0.5	1.00 g	1.00 g
CNCs-TDI	52.6	42.5	4.1	0.5	0.3	1.25 ± 0.01 g	1.24 g
CNCs-TDI-Eth	53.1	42.3	4.0	0.7	0.3	1.29 ± 0.01 g	1.30 g
CNCs-TDI-But	54.5	41.6	3.8	0.6	0.4	1.35 ± 0.01 g	1.34 g
CNCs-TDI-Hex	55.3	40.1	3.7	0.6	0.4	1.37 ± 0.01 g	1.37 g
CNCs-TDI-Oct	56.9	38.6	3.5	0.6	0.3	1.40 ± 0.01 g	1.41 g

**Table 2:** The thermal stability of the CNCs before and after surface modification.

Sample	T_o_ (°C)	T_d_ (°C)	Char Fraction (%)
CNCs	240 ± 2	250 ± 0	17.6 ± 0.7
CNCs-TDI	292 ± 0	351 ± 0	9.3 ± 0.2
CNCs-TDI-Eth	223 ± 0	319 ± 0	11.7 ± 0.1
CNCs-TDI-But	224 ± 0	317 ± 0	9.1 ± 0.0
CNCs-TDI-Hex	227 ± 1	318 ± 0	7.9 ± 0.4
CNCs-TDI-Oct	228 ± 1	320 ± 0	8.3 ± 0.0

**Table 3:** The DMA and TGA results for the reinforced PBS compared to the neat one.

Sample	DMA			TGA	
	E’_−60__°C_ (GPa)	E’_25__°C_ (GPa)	T_g_ (°C)	T_o_ (°C)	T_d_ (°C)
PBS	1.83 ± 0.10	0.37 ± 0.03	−17.3 ± 0.4	369 ± 2	391 ± 1
PBS + CNCs-TDI	2.02 ± 0.05	0.52 ± 0.03	−16.0 ± 1.0	368 ± 1	390 ± 0
PBS + CNCs-TDI-Eth	2.11 ± 0.04	0.54 ± 0.01	−17.3 ± 1.4	370 ± 0	392 ± 0
PBS + CNCs-TDI-But	2.27 ± 0.06	0.65 ± 0.03	−18.2 ± 0.4	370 ± 1	391 ± 1
PBS + CNCs-TDI-Hex	2.89 ± 0.09	0.77 ± 0.02	−17.5 ± 0.1	369 ± 0	391 ± 0
PBS + CNCs-TDI-Oct	2.99 ± 0.03	0.76 ± 0.02	−17.3 ± 0.1	370 ± 1	392 ± 1
